# Abnormal skeletal growth patterns in adolescent idiopathic scoliosis

**DOI:** 10.1186/1748-7161-8-S2-O34

**Published:** 2013-09-18

**Authors:** Houria Kaced, Hanene Belabbassi, Assia Haddouche

**Affiliations:** 1 Physical Medicine and Rehabilitation Department of University Hospital of Douera, Algiers, Algeria

## Background

Adolescent Idiopathic Scoliosis (AIS) occurs during a child's pubertal growth spurt. Although there is no clear consensus on the difference in body height between girls and boys with AIS and healthy controls, it is generally thought that the development and curve progression in children with AIS is closely associated with their growth rate. We proceeded to perform a prospective study on the anthropometric parameters of children with AIS.

## Purpose

Our aim is to compare the anthropometric parameters of children with AIS with those of a control group, using cross-sectional data set in comparison with the children's ages.

## Methods

A total of 431 children (258 girls and 173 boys, aged 9 to 16 years) were included in the study. Of the girls, 110 had AIS and 148 were healthy controls; of the boys, 49 had AIS and 124 were healthy controls. Clinical data and detailed anthropometric parameters were recorded. In the cross-sectional analysis, the groups of subjects were compared within different age groups ranging from 9 to 16 years old.

## Results

In the cross-sectional analysis, the girls with AIS were generally taller and heavier, than the healthy controls. Specifically, the girls with AIS were found to be significantly taller and heavier at the age of 12 years old, whereas the boys with AIS were significantly taller at the age of 14 years old than the healthy controls.

## Conclusions and discussion

The growth patterns in terms of height of children with AIS were significantly different from the healthy controls at the age of 12 for girls and at the age of 14 for boys.
